# Gastric Epithelial Expression of IL-12 Cytokine Family in *Helicobacter pylori* Infection in Human: Is it Head or Tail of the Coin?

**DOI:** 10.1371/journal.pone.0075192

**Published:** 2013-09-17

**Authors:** Fadi Al-Sammak, Thomas Kalinski, Sönke Weinert, Alexander Link, Thomas Wex, Peter Malfertheiner

**Affiliations:** 1 Department of Gastroenterology, Hepatology and Infectious Diseases, Otto-von-Guericke University, Magdeburg, Germany; 2 Department of Pathology, Otto-von-Guericke University, Magdeburg, Germany; 3 Department of Cardiology, Angiology and Pneumology, Otto-von-Guericke University, Magdeburg, Germany; 4 Medical Laboratory for Clinical Chemistry, Microbiology and Infectious Diseases, Department of Molecular Genetics, Magdeburg, Germany; Virginia Tech, United States of America

## Abstract

Recently, there has been a growing interest in an expanding group of cytokines known as “IL-12 family”. The so far gained knowledge about these cytokines, as crucial playmakers in mucosal immunity, has not yet been sufficiently investigated in the context of *Helicobacter pylori* infection. All genes encoding the monomeric components of these cytokines and their corresponding receptors were examined in gastric epithelial cell lines (AGS and MKN-28) after being infected with 4 *H. pylori* strains: BCM-300, P1 wild-type, and P1-derived isogenic mutants lacking cytotoxin-associated gene A (*cagA*) or virulence gene *virB7* (multiplicity of infection=50). Both infected and uninfected samples were analyzed after 24h and 48h using real-time quantitative polymerase chain reaction (RT-qPCR). Gene expression analysis demonstrated a strong upregulation of *IL23A* (encodes p19) by infection, whereas *IL23R, Epstein–Barr virus-induced gene 3* (*EBI3*), *IL6ST*, *IL12A*, and *IL27RA* were found to be expressed, but not regulated, or to a lesser extent. Transcripts of *IL12RB2, IL12B*, *IL12RB1*, and *IL27A* were not detected. Interestingly, P1 resulted in stronger alterations of expression than CagA mutant and BCM-300, particularly for *IL23A* (59.7-fold versus 32.4- and 6.7-fold, respectively in AGS after 48h, P<.05), whereas no changes were seen with VirB7 mutant. In a proof-of-principle experiment, we demonstrated epithelial-derived expression of IL-12, p19, and Ebi3 in gastric mucosa of gastritis patients using immunohistochemistry (IHC). Unlike IL-12 and Ebi3, increased immunostaining of p19 was observed in *H. pylori* gastritis. Herein, we highlight the potential role of gastric epithelial cells in mucosal immunity, not only because they are predominant cell type in mucosa and initial site of host-bacterial interaction, but also as a major contributor to molecules that are thought to be primarily expressed by immune cells so far. Of these molecules, p19 was the most relevant one to *H. pylori* infection in terms of expression and localization.

## Introduction

**Figure 1:**
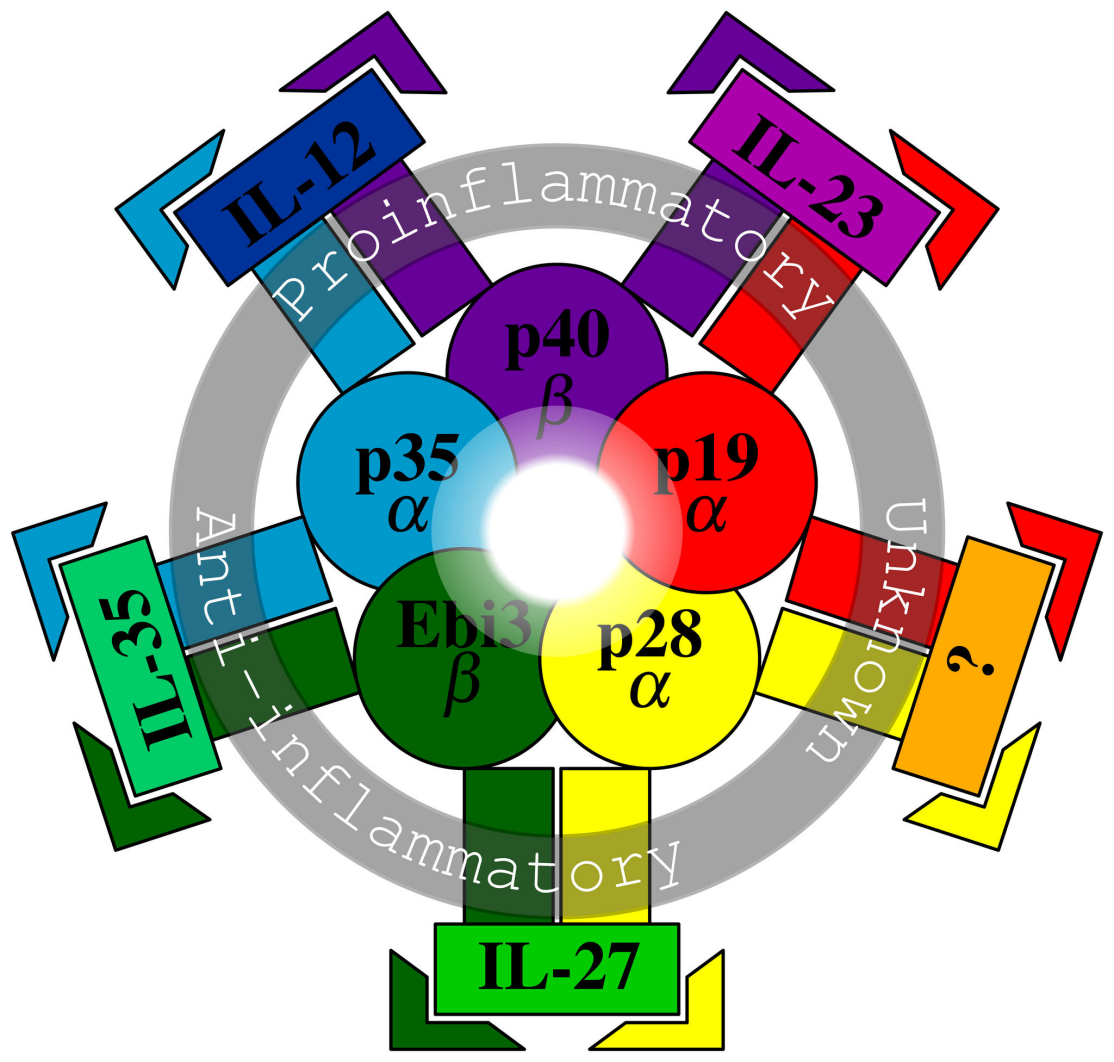
Schematic diagram of IL-12 family. The diagram illustrates the currently known 5 chains (the innermost zone), composite cytokines with their proposed functions (the middle zone), and their receptors (the outermost zone). The five color-coded chains are either α chains (p19, p35, and p28) or β chains (p40 and Ebi3). Each functional cytokine is made of one α and one β chain. IL-12 (p35/p40) is a proinflammatory Th1 activator and stabilizer. Similarly, IL-23 (p19/p40) is a proinflammatory Th1 activator and Th17 stabilizer. On the other hand, IL-27 (p28/Ebi3) is an immunoregulatory cytokine mainly with an anti-inflammatory capacity. IL-35 (p35/Ebi3) is again anti-inflammatory and secreted by Treg cells. Therefore, the four cytokines with their divergent functions can promote a representative model of the immunological spectrum ranging from inflammation to tolerance. The unknown cytokine is a theoretical one and symbolizes the possibility of unraveling other unknown family members in the future. Each chain (p19, p40, p35, Ebi3, p28) binds to its specific receptor (IL23R, IL12RB1, IL12RB2, IL6ST, and IL27RA, respectively) and is coded with the same color of the corresponding chain (receptor names are not shown). Typically, a composite cytokine would require a β chain receptor for high affinity binding and an α chain receptor for signal transduction. For alternative terminology of IL-12 family members refer to Table 1.

The pathological spectrum associated with *Helicobacter pylori* infection is broad and ranges from asymptomatic chronic gastritis to gastric cancer. The outcome is defined by complex host-pathogen interactions. On the host part, a balance between gastric mucosal immunity and tolerance is maintained by intricate network of signaling pathways. A large body of evidence suggests that Th1, and most recently Th17, mediate a proinflammatory response against *H. pylori* infection towards clearance, whereas regulatory T (Treg) cells mediate anti-inflammatory activities towards tolerance and persistence. This balanced functional dichotomy of the immune response is epitomized by an emerging and expanding family of cytokines called “IL-12 family” (reviewed in 1). It comprises two proinflammatory cytokines (IL-12 and IL-23) and two mainly anti-inflammatory cytokines (IL-27 and IL-35). They are all heterodimeric cytokines and have a common remarkable feature of “chain sharing”. Each of the currently known five chains can be either α chain (p19, p35, and p28) or β chain (p40 and Ebi3), and each binds to a specific receptor. A functional composite cytokine is typically made of shared α and β chains. A summarizing diagram of these chains, composite cytokines, and their receptors is provided in Figure 1. Similar to IL-12 for Th1 cells, IL-23 can serve to expand and stabilize Th17 responses [2]. It has also been reported that IL-23 can activate Th1 memory cells [3] and inhibit accumulation of Treg cells in the intestine [4]. IL-27 was initially identified to be a proinflammatory cytokine by initiation of Th1 response [5]; however, subsequent studies revealed its pleiotropic nature mainly with an anti-inflammatory capacity as reviewed in 6. Finally, the most recent member of the family is IL-35 [7], which is known to be a potent inhibitor and secreted by Treg cells [8,9].

Virulent strains of *H. pylori* are associated with more severe inflammation of the gastric mucosa. The cytotoxin-associated gene pathogenicity island (*cag* PAI) encompasses 32 genes including *cagA*, which encodes CagA protein [10]. The other genes encode a type IV secretion system (T4SS), a sort of a molecular syringe to translocate CagA and other molecules into the eukaryotic cells (reviewed in 11). The translocation and phosphorylation of CagA are followed by activation of NF-κB, induction of the proinflammatory cytokine IL-8, and remodeling of the gastric epithelial cytoskeleton [12,13]. Other factors, not encoded by cag PAI, have also been linked with more severe clinical outcome like VacA (a secretory multi-functional toxin), OipA, and DupA.

In recent years, there has been an increasing amount of literature on IL-12 family using different models and approaches. Apart from the fact that the majority of these studies were out of *H. pylori* context, their results should be interpreted with caution because of unawareness of the heterodimeric structure and frequency of chain sharing. For instance, p40^-/-^ mice lack both IL-12 and IL-23; consequently, any observed phenotype might be due to lack of one or both of these cytokines. Likewise, the approach of using p40 neutralizing antibodies is less straightforward and needs to be re-evaluated. The unique approach of this study is the complete analysis of IL-12 family, distilled to its five currently known monomeric chains with their receptors, using real time quantitative PCR (RT-qPCR), to gain basic insight into the influence of different virulent strains of *H. pylori* on the expression of all studied transcripts in AGS and MKN-28 cell lines as a gastric epithelial cell model. To evaluate the epithelial expression of IL-12 family in gastric mucosa at the protein level, we performed immunohistochemical staining of gastric biopsies from gastritis patients with and without *H. pylori* infection. 

## Materials and Methods

### Ethics Statement

Gastric biopsies were obtained from patients who underwent upper gastrointestinal endoscopy at the Department of Gastroenterology, Hepatology and Infectious Diseases, Otto-von-Guericke University in the frame of ERA-Net PathoGenoMics project. The study protocol was approved by Institutional Review Board at Otto-von-Guericke University, and all subjects provided a written informed consent.

### Cell Lines and *H. pylori* Strains

Human gastric epithelial cell lines were purchased from American Type Culture Collection (ATCC) and the Japanese Cancer Research Resources Bank: AGS (CRL-1739) and MKN-28 (JCRB-0253), respectively. Gastric cancer cells were maintained in 75cm^2^ cell culture flasks (NUNC GmbH, Wiesbaden, Germany) in a cell incubator at 37°C and 5% CO_2_. All cell lines were cultivated using RPMI-1640 containing 10% fetal calf serum (FCS), 100U/mL Penicillin, 100µg/mL streptomycin, and 100µg/mL gentamycin. All reagents were purchased from PAA (Cölbe, Germany). Infection studies were implemented using a previously described *H. pyloricagA* ^+^ and *vacA* s1/m1 wild-type P1 strain along with its two isogenic mutants: P1∆*cagA* and P1∆*virB7*; we called them “M1” and “M2”, respectively. VirB7 protein is crucial for the assembly of the T4SS and its lack would render the whole T4SS non-functional [14]. Another strain was included in this study which is *H. pylori* ATCC® BAA-1606™ (*cagA* ^+^, *vacA* s1/m1, *babA2*, *oipA* functional, and designated BCM-300), the same strain was previously used to challenge healthy volunteers in a vaccine trial against *H. pylori* [15]. These strains were cultivated on selective agar plates (bioMérieux, Marcy l’Etoile, France) under microaerophilic conditions at 37°C for 2 days. 2x10^5^ cells of AGS and MKN-28 were seeded in complete medium in 6-well plates (Greiner Bio-One GmbH, Frickenhausen, Germany) and cultivated for 24h. After washing cells twice with medium without FCS and antibiotics, cells were infected with *H. pylori* strains at a multiplicity of infection (MOI) of 50 suspended in antibiotic-free medium containing 10% FCS. Bacterial suspensions were adjusted based on optical density at 535nm and 550nm for BCM-300 and other strains, respectively (OD=1 corresponds to 1x10^9^ bacteria). To ensure functional active bacteria, suspensions were microscopically inspected for shape and motility. Uninfected cells were included as controls. All cells were cultivated and harvested for RNA extraction after 24h and 48h. Likewise, another experiment was performed in which only BCM-300 strain was used to infect AGS and MKN-28 cells in a 6-well plate either directly or indirectly by using a 0.22µm filter cap to separate the bacteria from the cells, while allowing its secretory factors to pass through. All cells were also harvested for RNA extraction after being cultivated for 24h and 48h. Both experiments were replicated 4 times.

### Real-Time Quantitative PCR (RT-qPCR)

Total RNA of cell lines was isolated by a column-based extraction method using RNeasy kit™ (Qiagen, Hilden, Germany) including an additional DNAse-treatment to remove genomic DNA in accordance to the manufacturer’s protocol. Finally, RNA was eluted in 40µL RNase-free water and 5µl out of each sample were used to measure the concentration via UV-spectroscopy and to examine the integrity of RNA by 1% gel electrophoresis. This was followed by cDNA synthesis of equal amounts of RNA (500ng) from each sample by using random hexamers and stored at -80°C for later analysis by RT-qPCR. Intron-spanning/flanking primers were designed to avoid amplification of genomic DNA. Gradient PCR was performed to determine optimal annealing temperatures. PCR efficiencies were determined by dilution curves. Only primer sets with an amplification factor of 1.9 or more were selected for the analysis. In addition to IL-12-related genes, we investigated the expression of *IL8*, for being well documented to be induced by gastric epithelial cells upon infection with *H. pylori* [16]. All primer details are provided in Table 1. PCR reaction setup and cycling conditions were set according to QuantiTect™ SYBR Green kit (Qiagen, Hilden, Germany) with reaction volume of 30µL, primer concentration at 0.3µM, and for 40 cycles (Table 1). All experimental replicates were analyzed in one 96-well plate along with negative (NTC) and positive controls. Quantification cycles (Cqs) of the studied transcripts were measured using CFX96-Cycler™ (BioRad, Munich, Germany). Melting curve analysis of the PCR products was also performed. Expression was normalized against two reference genes (*ACTB* and *RPL29*) in light of recent data [17]. Normalized relative quantities (NRQs) and fold changes were calculated using the geNorm algorithm [18] with the aid of R based ”SLqPCR” package [19]. Stability index (M) of the two reference genes was 0.5 and 0.7 for the two aforementioned cell line experiments, respectively.

**Table 1:** Primers used for RT-qPCR analysis.

Transcript	Primer sequence^1^	RefSeq^2^	AnT^3^	Amplicon	Exons^4^	Intron	E^5^
	**(from 5’ to 3’**)		**(°C**)	**size (bp**)		**size (bp**)	
*ACTB* (a reference gene that encodes a cytoskeletal actin)							
fwd	AAGGCCAACCGCGAGAAGATG	NM_001101	57	100	f (3,4)-6	441	2
rev	CAGAGGCGTACAGGGATAGCAC						
*RPL29* (a reference gene that encodes a ribosomal protein in the 60S subunit)							
fwd	GCCAAGTCCAAGAACCACAC	NM_000992	57	133	f (2,4)-4	1233	2
rev	CAAAGCGCATGTTCCTCAGG						
*IL8* (encodes a protein in the CXC chemokine family called IL-8)							
fwd	CTTCCTGATTTCTGCAGCTCTG	NM_000584	57	193	f (1,3)-4	1090	2
rev	GAGCTCTCTTCCATCAGAAAGC						
*IL23A* (encodes p19 that pairs with p40 to form IL-23)							
fwd	**GACACAT**GGATCTAAGAGAAGAG	NM_016584	57	109	s (**1,3**)-4	385	1.95
rev	**AA**CTGACTGTTGTCCCTGAG						
*IL23R* (encodes IL23A receptor which is required, along with IL12RB1, for signaling of IL-23 via p19)							
fwd	TCCTGTGAAATGAGATACAAGGC	NM_144701	59	104	f (6,7)-11	12518	1.95
rev	GGCTCCAAGTAGAATTCTGACTG						
*EBI3* (*IL27B*, encodes Ebi3 that pairs with p28 to form IL-27)							
fwd	AGCTTCGTGCCTTTCATAACAG	NM_005755	57	140	f (3,4)-5	1359	2
rev	AGTGAGAAGATCTCTGGGAAGG						
*IL6ST* (*gp130*, *oncostatin* ** *M* ** *receptor*, *CD130*, encodes a signal transducer that is shared by many cytokines)							
fwd	ACTGTCCAAGACCTTAAACCT	NM_001190981	57	145	f (7,8)-17	3110	1.98
rev	AGAAACTTGGTGCTTTAGATGG						
*IL12A* (*NKSF1*, *CLMF1*, encodes p35 that pairs with p40 and Ebi3 to form IL-12 and IL-35, respectively)							
fwd	AATGTTCCCATGCCTTCACC	NM_000882	59	110	f (2,3)-7	2699	2
rev	CAATCTCTTCAGAAGTGCAAGGG						
*IL12RB2* (encodes a protein that, along with IL12RB1, forms a high affinity receptor for IL-12 via p35)							
fwd	CCTCTTCACTTCCATCCACATTC	NM_001559	57	137	f (5,6)-16	1202	1.96
rev	AAGCAGTACCAGTCCCTCATC						
*IL12B* (*NKSF2*, *CLMF2*, encodes p40 that pairs with p35 and p19 to form IL-12 and IL-23, respectively)							
fwd	**GGACATCA**TCAAACCTGACC	NM_002187	56	123	s (5,6)-8	1412	2
rev	AGGGAGAAGTAGGAATGTGG						
*IL12RB1* (encodes a low affinity protein that, along with IL12RB2, forms IL-12 receptor)							
fwd	GCTGTACACTGTCACACTCTG	NM_005535	57	132	f (4,5)-17	3176	1.9
rev	AACTTGGACACCTTGATGTCTC						
*IL27A* (*IL30*, encodes p28 that pairs with Ebi3 to form IL-27)							
fwd	CTCCCTGATGTTTCCCTGAC	NM_145659	57	145	f (3,4)-5	1560	2
**rev**	TCCTCTCCATGTTGGTCCAG						
*IL27RA* (*WSX1*, *CRL1*, *TCCR*, *zcytor1*, encodes a glycosylated transmembrane protein)							
fwd	AGATGTGTGGGTATCAGGGAAC	NM_004843	57	102	f (6,7)-14	3364	2
rev	ACTTTGTAGCTCACCTGCAC						

^1^ Letters in bold represent the spanning part of the sequence

^2^ RefSeq; the NCBI database of reference sequences (http://www.ncbi.nlm.nih.gov)

^3^ AnT; annealing temperature of the qPCR reaction (95^15^, 94´^15^, AnT´^30^, 72´^30^, 72^5^)

^4^ Indicates which exons flanked (f) or spanned (s) by a primer set followed by the total number of exons for that transcript, e.g., *ACTB* primer set flanks exon 3 and exon 4 of 6 total exons

^5^ Amplification efficiency as determined by dilution curve

### Immunohistochemistry (IHC)

The formalin-fixed, paraffin-embedded specimens of gastric biopsies from antrum and corpus of gastritis patients with and without *H. pylori* infection (5 of each) were collected from the archive of the Pathology Department. Cases were classiﬁed as *H. pylori* positive or negative only if all of the three tests (urease, histology, and microbiological culture) were positive or negative, respectively. Biopsies of atrophic gastritis and/or intestinal metaplasia were excluded. IHC was performed with avidin–biotin complex immunostaining method using automated Nexes slide stainer (Ventana Medical Systems, Tucson, AZ, USA). Sections of the specimens (3µm thick) were mounted on SuperFrost Plus glass slides (Menzel, Braunschweig, Germany), dewaxed by immersion in xylene, and rehydrated by descending concentrations of ethanol. Heat-mediated antigen retrieval was performed for 30sec at 120°C using DakoCytomation Pascal pressure chamber (DakoCytomation, Glostrup, Denmark) in 1mM citrate pH 6.0 for IL-12, or in 1mM EDTA pH 8.0 for both p19 and Ebi3. Slides were incubated at room temperature (RT) with anti-IL-12 antibody (ab9992; Abcam, Cambridge, UK), anti-p19 antibody (ab45420; Abcam), anti-Ebi3 antibody (ab83896; Abcam), diluted to 1/50, 1/100, 1/200, for 30min, 1h, and 1h, respectively. The reactions were visualized using 3,3’-diaminobenzidine (DAB) for IL-12 and Ebi3 or enhanced alkaline phosphatase (AP) for p19. The slides were counterstained with hematoxylin, embedded in mounting medium, and cover-slipped. Microscopic evaluation of the staining results was performed using a Zeiss Axioplan microscope (Zeiss, Oberkochen, Germany) and an Olympus DP26 digital camera (Olympus, Tokyo, Japan). Tissue sections from human astrocytoma tumor, normal kidney, and normal spleen were used as tissue positive controls for anti-IL-12, anti-p19, and anti-Ebi3 antibodies, respectively. For negative controls, primary antibody was replaced with PBS buffer and did not reveal specific signals (data not shown).

### Immunofluorescence (IF)

The same serial sections from gastritis cases were used for IF staining of p19. Dewaxing, rehydration, and heat-mediated antigen retrieval were performed the same way as in IHC for p19. Slides were blocked with PBS containing 10% FCS for 1h, followed by 1h incubation at RT with anti-19 antibody (ab45420; Abcam) diluted to 1/100. For negative controls, primary antibody was replaced by PBS buffer. Slides were then washed three times and incubated with anti-rabbit Cy3-conjugated F(ab')2 fragments (Sigma-Aldrich, Inc., St. Louis, MO, USA) at a dilution of 10µg/mL for another 1h, followed by additional washing, 4',6-diamidino-2-phenylindole (DAPI) staining, and mounting for fluorescence microscopy.

### Microscopy and Image Processing

Microscopy was performed with Zeiss Axiovert 200m-based epi-fluorescence wide-field imaging system (Carl Zeiss MicroImaging GmbH, Jena, Germany), equipped with a fluorescence filter set; Zeiss No. 49, HQ-Cy3.5 (EX 565/30, BS 585, EM 620/60) (Chroma Technology Corp., Bellows Falls, VT, USA), a scanning stage (Märzhäuser Wetzlar GmbH & Co. KG, Wetzlar, Germany), and an AxioCam MRm camera (Carl Zeiss MicroImaging GmbH). Images were acquired with Plan-Neofluar 10x/0.30 Ph1 for stitched overviews, and EC, Plan-Neofluar 40x/0.75 Ph2 for higher magnified regions of interest (ROI). To prove the comparability between the sample section and its adjacent control section, all acquisition parameters, i.e., acquisition time, camera gain, and light intensity were kept constant. Acquisition and device control were performed using AxioVision 4.8.1 software (Carl Zeiss MicroImaging GmbH).

### Statistical Analysis

Fold changes were logarithmically transformed before implementing statistical analysis. For comparisons among different *H. pylori* strains, non-parametric Kruskal-Wallis test was performed. Pairwise comparisons were then implemented using Mann-Whitney *U* test with multiple testing correction using Benjamini-Hochberg (BH) procedure. Correlation analysis was performed using non-parametric Spearman’s *rho* test. For all tests, two-tailed P-values <.05 were considered significant. All statistical tests were conducted using R programming language [20] and graphs were generated with the aid of R based “ggplot2” package [21]. 

## Results

### Gene Expression Profile of IL-12-Related Molecules in Gastric Epithelial Cells

**Figure 2:**
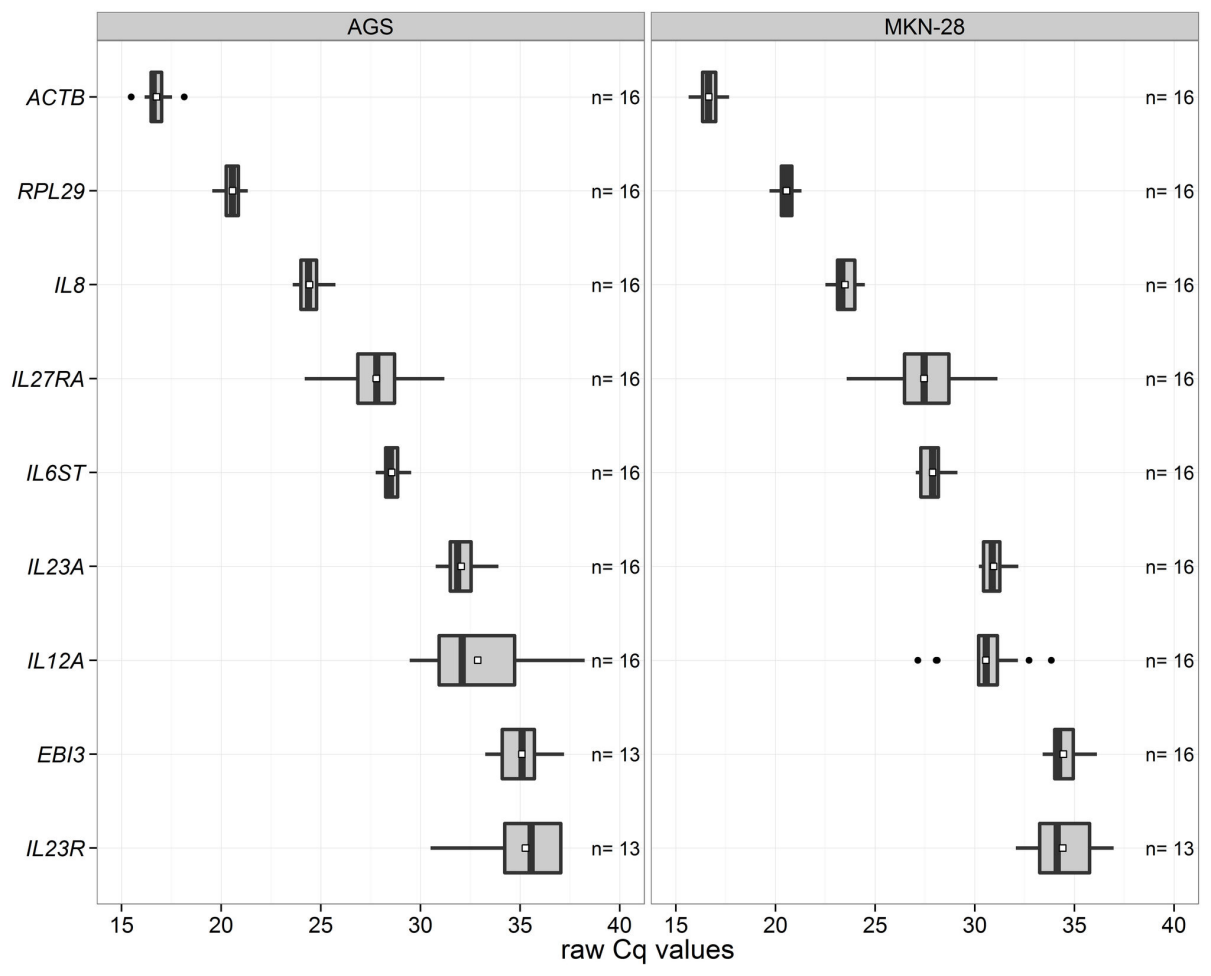
Boxplots of raw Cqs of the detected transcripts by RT-qPCR in non-infected AGS and MKN-28. Each box represents the lower quartile, the median, and the upper quartile score (vertical lines of the box). Means (small white squares) and outliers are also shown (black dots). Expression levels of detected transcripts were ordered in AGS cell line from highest (*ACTB*) to lowest (*IL23R*) based on their median values. Similar ranking order was seen in MKN-28 cells except for the least four abundant transcripts: *IL23A*, *IL12A*, *EBI3*, and *IL23R*.

Basal gene expression levels of IL-12-related molecules (Figure 1) were studied in two gastric epithelial tumor cell lines (AGS and MKN-28) using RT-qPCR. To examine their abundance in the two cell lines, raw Cq values of these transcripts along with other three transcripts (*ACTB*, *RPL29*, and *IL8*) in uninfected samples (controls) were plotted (Figure 2). As illustrated, six of the IL-12-related genes were expressed in both cell lines at levels below *ACTB*, *RPL29*, and *IL8*. Notably, *IL27A*, *IL12B*, *IL12RB1*, and *IL12RB2* transcripts were not detected in epithelial cells. Generally, both cell lines showed a similar expression pattern for all investigated transcripts (Figure 2).

### Gene Expression of IL-12-Related Molecules in Context of *H. pylori* Infection

**Figure 3:**
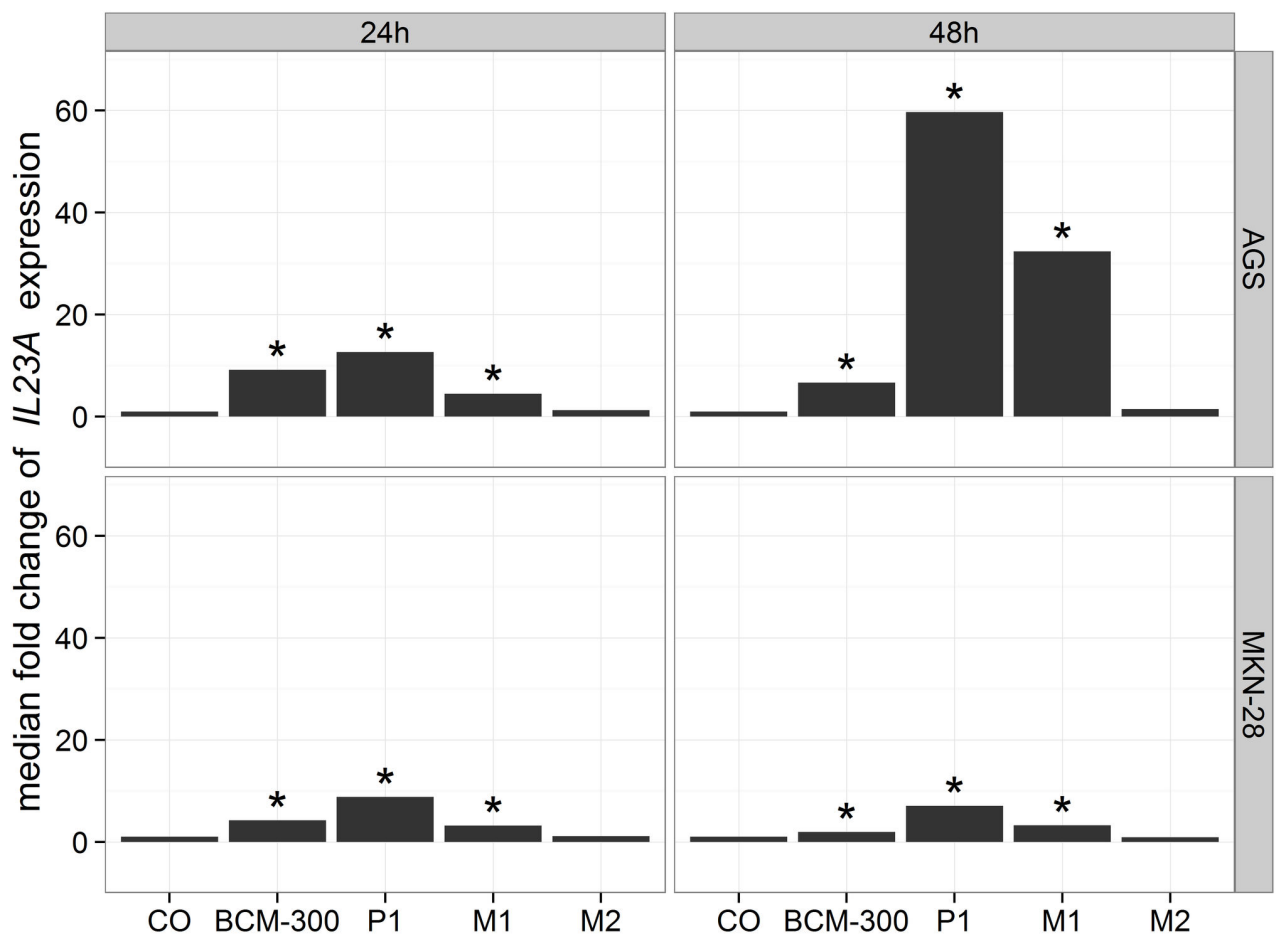
Barcharts of *IL23A* median fold change by RT-qPCR in AGS and MKN-28 (n=4). BCM-300, P1, M1, and M2 are *H. pylori* strains. Both BCM-300 and P1 (wild-type) are CagA strains, whereas M1 and M2 are P1-derived isogenic mutants lacking CagA and VirB7, respectively. Generally, higher expression levels were seen in AGS more than MKN-28 cells. Significant upregulation of *IL23A* was seen in both cells with BCM-300, P1 (the highest), and M1 strain (P<.05), whereas no alteration was seen after M2 infection (non-functional T4SS) similar to that of the non-infected control samples (CO).

To evaluate the effect of *H. pylori* infection on expression of the investigated genes, we analyzed fold changes of the detected transcripts in AGS and MKN-28 cells after infection with different strains of *H. pylori* for 24h and 48h. Additionally, *IL8* was included as an internal control to our study for being constitutively expressed by gastric epithelial cells and induced upon *H. pylori* infection as well. Both CagA positive strains BCM-300 and P1, as well as P1-derived isogenic mutant M1, showed a consistent induction of *IL8* transcript levels after 24h and 48h, whereas M2 mostly did not induce *IL-8* mRNA levels (Table 2). The highest increase was seen in MKN-28 cells after 24h infection with P1 strain (median of 17.5-fold). Concerning IL-12-related genes, *IL23A* showed a pronounced expression pattern similar to that of *IL8*. In general, transcript levels of *IL23A* in AGS were higher than those in MKN-28 cells. The highest expression levels in AGS were observed after 48h of infection with P1 and M1 strains (medians of 59.7-fold and 32.4-fold, respectively), lower levels were attained with BCM-300 infection (median of 6.7-fold), whereas no induced expression was seen with M2 strain (Figure 3). The upregulation of *IL6ST* and *EBI3* was less pronounced after infection compared to that of *IL8* and *IL23A* (Table 2). On the other hand, *IL23R* was found to be downregulated in MKN-28 cells after 48h of infection with P1 and its two isogenic mutants (P<.05), but not with BCM-300 strain. Of note, *IL27RA* expression showed a decreasing trend over time, but this observation did not reach a significant level (Table 2). Finally, no significant alterations were found in *IL12A* after infection in both cell lines (P>.05). A consistent pattern was observed by comparing fold changes of most transcripts in P1 with those of its two isogenic mutants. Infection by P1 resulted in more and stronger alterations than M1, whereas M2 was mostly similar to controls (without *H. pylori*). Overall, BCM-300 strain demonstrated an expression pattern that was more or less similar to that of P1 and M1 (Table 2).

**Table 2:** Median and median absolute deviation (MAD) of fold change of IL-12-related transcripts by RT-qPCR in AGS and MKN-28 cells after infection with different *H. pylori* strains (n=4).

		AGS				MKN-28			
		24h		48h		24h		48h	
Transcript	**Strain**	*median*	*MAD*	*median*	*MAD*	*median*	*MAD*	*median*	*MAD*
***IL8***		*		*		**		*	
	BCM-300	6.8*	5.3	3.5*	1.3	4.6*	2.5	3.1*	1.6
	P1	9.9*	9.7	10.3*	12.4	17.5*	20.2	7.1*	5.2
	M1	2.6*	0.7	8.4*	9.4	6.6*	2.3	2.8*	1.7
	M2	1.7	0.7	1.5	0.6	1.5*	0.1	1.5	0.9
***IL23A***		**		*		**		**	
	BCM-300	9.2*	2.2	6.7*	2.2	4.2*	2.6	2*	0.4
	P1	12.7*	11.9	59.7*	85.3	8.8*	6.1	7.1*	6.2
	M1	4.5*	1.7	32.4*	37.1	3.2*	0.7	3.3*	1.8
	M2	1.3	0.4	1.5	1.3	1.1	0.3	0.9	0.4
***IL23R***								*	
	BCM-300	3.5	4.3	1	0.8	1.4	0.8	0.7	0.2
	P1	3.5	2.4	0.4	0.3	1.5	1.4	0.4*	0.3
	M1	0.9	1.2	0.2	0.2	0.8	0.7	0.3*	0.2
	M2	0.9	1.2	1.2	1.0	1.2	0.1	0.4*	0.1
***EBI3***						*		**	
	BCM-300	1.8	0.8	1.6	0.4	2.6*	0.5	1.7*	0.5
	P1	2.2	1.6	1.4	0.8	2	1.6	2.4*	1.3
	M1	0.8	0.1	2.2	1.4	2.5*	0.2	1.9*	0.6
	M2	0.4	0.2	1.1	0.8	1.2*	0.3	0.8	0.2
***IL6ST***									
	BCM-300	1.3*	0.1	1.8*	0.5	1.2	0.4	1.6	0.2
	P1	1.5	0.8	1.7*	0.2	1.3	0.3	1.8	0.5
	M1	1.2*	0.2	1.8	1.1	1	0.2	2.5	0.4
	M2	1.2	0.7	1	0.2	0.6	0.2	1.1	0.5
***IL12A***									
	BCM-300	0.8	0.3	0.7	0.3	1.2	0.4	0.9	0.1
	P1	0.7	0.1	0.7	0.2	1.6	0.5	0.8	0.4
	M1	0.8	0.2	0.9	0.2	1	0.5	0.8	0.2
	M2	0.9	0.1	0.7	0.1	1.1	0.2	0.7	0.1
***IL27RA***									
	BCM-300	1.2	0.2	1	0.1	1.2	0.1	0.8	0.1
	P1	1.3	0.6	0.6	0.2	1.2	0.2	0.7	0.2
	M1	0.8	0.2	0.7	0.5	1.1	0.3	0.7	0.2
	M2	1	0.4	1	0.3	0.9	0.2	0.8	0.1

^1^ *P<.05, ** <.01, *** <.001

^2^ Asterisks of significance levels in the row of the transcript name correspond to the global Kruskal-Wallis test among different strains for that particular transcript, whereas asterisks in the row of the strain name correspond to Benjamini-Hochberg (BH) adjusted P values of pair-wise contrasts with Mann-Whitney *U* test

^3^ BCM-300, P1, M1, and M2 are *H. pylori* strains. Both BCM-300 and P1 (wild-type) are CagA strains, whereas M1 and M2 are P1-derived isogenic mutants lacking CagA and VirB7, respectively.

### BCM-300-Induced Alterations Depend on Direct Contact with Gastric Epithelial Cells

Using a 0.22µm filter cap we studied whether direct contact between bacteria and epithelial cells is critical for the expression of IL-12-related transcripts. As summarized in Table 3, most altered gene expression patterns were found to be dependent on the direct contact between cells and bacteria. Application of the filter cap prevented most of these alterations; however, some of the alterations remained significant even after application of the filter cap (e.g., *IL23A*; 1.6-fold, P<.05), but the magnitude of the fold change was minute compared to that observed with the direct contact (15.1-fold, P<.05).

**Table 3:** Median and median absolute deviation (MAD) of fold change of normalized relative quantities (NRQs) of IL-12-related transcripts by RT-qPCR in AGS and MKN-28 cells after infection with *H. pylori* BCM-300 strain (n=4).

		AGS				MKN-28			
		24h		48h		24h		48h	
Transcript	**Sample**	*median*	*MAD*	*median*	*MAD*	*median*	*MAD*	*median*	*MAD*
*IL8*		**		**		*		*	
	HP+Fi	1.8*	0.1	3.7*	1.0	1.2	0.1	3.8*	1.9
	HP	24.2*	5.1	20.4*	4.6	22.1*	2.9	12.8*	5.8
*IL23A*		**		*		*			
	HP+Fi	1.6*	0.2	1.6	1.0	1.3	0.2	1.6	0.8
	HP	15.1*	2.9	3.9*	1.6	7.6*	3.2	5.8	2.3
*IL23R*									
	HP+Fi	0.6	0.3	0.5	0.2	1.4	0.3	0.1	0.1
	HP	1	0.4	0.6	0.5	1.3	0.9	0.5	0.5
*EBI3*		**		*		*			
	HP+Fi	0.3	0.3	1.9	1.3	0.4*	0.3	1	0.4
	HP	4.6*	3.2	15.3*	1.8	2.8	2.1	1.7*	0.7
*IL6ST*		**		*		*		*	
	HP+Fi	1.3*	0.2	2.4*	0.6	0.8	0.1	1.9*	0.7
	HP	3.3*	1.2	3.7*	1.8	2*	0.6	2.8*	1.6
*IL12A*									
	HP+Fi	0.8	0.1	0.7*	0.3	1	0.2	0.4*	0.2
	HP	0.5	0.2	1	0.7	0.5	0.1	0.5	0.2
*IL27RA*									
	HP+Fi	0.8	0.2	0.6	0.2	0.8*	0.1	0.8*	0.1
	HP	0.9	0.2	0.3*	0.2	1	0.5	0.5	0.2

^1^ *P<.05, ** <.01, *** <.001

^2^ Asterisks of significance levels in the row of the transcript name correspond to the global Kruskal-Wallis test among samples for that particular transcript, whereas asterisks in the row of the sample correspond to Benjamini-Hochberg (BH) adjusted P values of pair-wise contrasts with Mann-Whitney *U* test

^3^ “HP+Fi”; *H. pylori* BCM-300 strain with a 0.22µm filter cap, “HP”; the same strain without a filter cap

### Exploring the Relationship between *IL23A* and Other Transcripts after Infection with Different *H. pylori* Strains in Gastric Epithelial Cells

**Figure 4:**
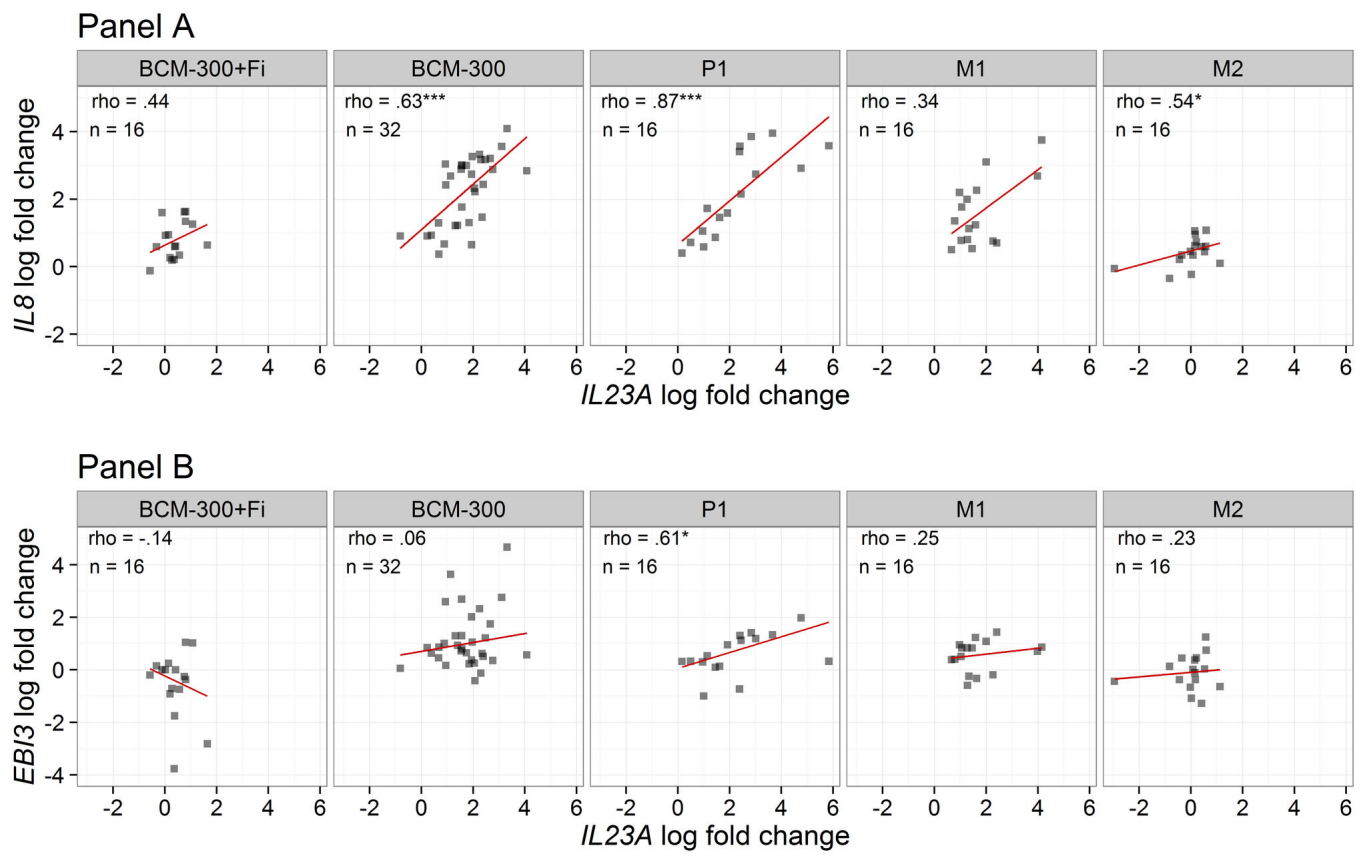
Correlation of *IL23A* with *IL8* and *EBI3* after infection with different *H. pylori* strains. Scatterplots of log-transformed fold changes of transcripts by RT-qPCR illustrate the relationship of *IL23A* with both *IL8* (panel A) and *EBI3* (panel B) transcripts after infection with different *H. pylori* strains using pair-wise Spearmen’s *rho* correlations. Robust regression line using an M estimator was plotted as well (red line). BCM-300, P1, M1, and M2 are *H. pylori* strains. Both BCM-300 and P1 (wild-type) are CagA strains, whereas M1 and M2 are P1-derived isogenic mutants lacking CagA and VirB7, respectively. BCM-300+Fi is infection with *H. pylori* BCM-300 bacteria after application of 0.22µm filter cap to prevent any physical contact with the cells, while permitting their secretory factors to pass through. Asterisks represent the significance levels (*P<.05, **<.01, ***<.001).

In order to discover important relationships between *IL23A* and other studied transcripts we implemented a Spearman’s correlation analysis of the logarithmically transformed fold changes of the transcripts. Data are exemplarily illustrated in Figure 4 and summarized in Table 4. The correlation between *IL23A* and *IL8* as illustrated in Figure 4A was the highest with P1 and BCM-300 strains (ρ=.87 and ρ=.63, respectively, P<.001) and less pronounced with M2 (ρ=.54, P<.05), whereas this correlation was lost when CagA protein is not expressed in M1 isogenic strain, or after application of the filter cap (P>.05). Remarkably, *IL23A* was positively correlated with *EBI3* after infection with P1 strain only (P<.05, Figure 4B). As shown in Table 4, the correlation between *IL23A* and *IL6ST* was only seen after infection with BCM-300 and P1 strains (P<.05). On the other hand, no correlation was found between *IL23A* and *IL27RA*.

**Table 4:** Spearman’s *rho* correlation table between *IL23A* transcript and other detected transcripts of both *IL8* and IL-12-related genes after infection with different *H. pylori* strains regardless of cell type or duration of infection.

	Strain	*IL8*	*EBI3*	*IL23R*	*IL6ST*	*IL27RA*	n
***IL23A***	BCM-300+Fi	.44+	-.14	-.33	.44+	-.24	16
	BCM-300	.63***	.06	-.15	.42*	.15	32
	P1	.87***	.61*	.21	.58*	.14	16
	M1	.34	.25	-.07	.12	-.22	16
	M2	.54*	.23	.32	-.1	-.29	16

^1^ +P<.1, *P<.05, ** <.01, *** <.001

^2^ “BCM-300+Fi”; *H. pylori* BCM-300 strain with a 0.22µm filter cap

### Immunostaining of IL-12, p19, and Ebi3 in Human Gastric Mucosa

**Figure 5:**
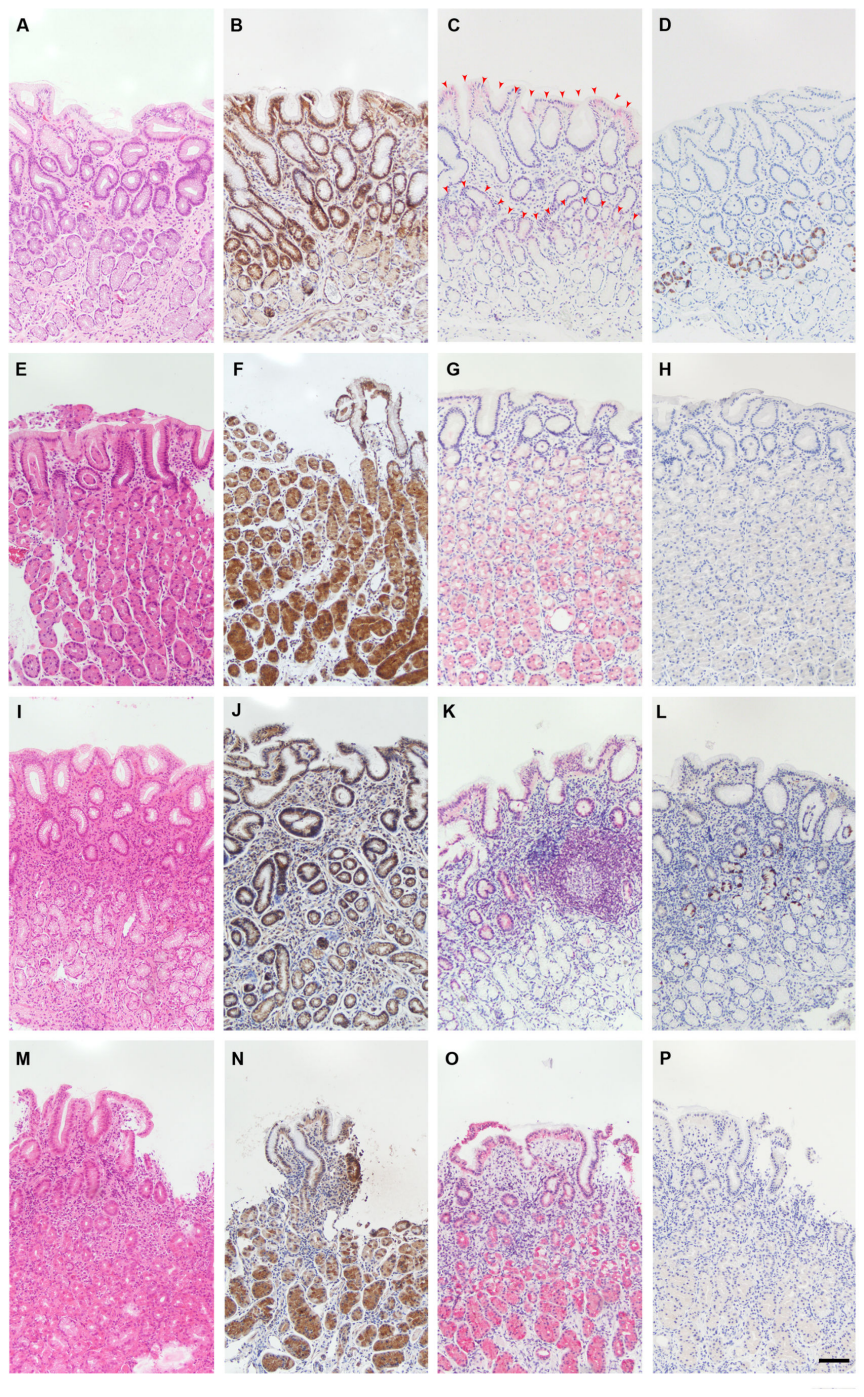
Immunohistochemical analysis of IL-12, p19, and Ebi3 in human gastric mucosa. Microscopic images from a typical case of *H. pylori* negative gastritis are shown in A-D (antrum) and E-H (corpus). The antrum mucosa (A) contains few immune cells (hematoxylin-eosin). Both immune and epithelial cells were stained with IL-12 in the antrum mucosa (B). Immunostaining of p19 was detected in both immune and epithelial cells with a tendency to be more localized in the superficial epithelial cells and glandular neck zone of the antrum mucosa (C; arrowheads). Immunostaining of Ebi3 was exclusively found in the glandular neck zone of the antrum mucosa (D). The corpus mucosa (E) contains also few immune cells and is rich in parietal cells (hematoxylin-eosin). Both immune and epithelial cells of the corpus mucosa were stained with IL-12 (F). Immune cells, foveolar epithelium, and corpus glands with parietal cells were all immunostained with p19 (G). No Ebi3 staining was detected in the corpus mucosa (H). Microscopic findings in *H. pylori* gastritis are shown in I-L (antrum) and M-P (corpus). The antrum mucosa in *H. pylori* gastritis (I) contains higher amounts of immune cells (hematoxylin-eosin). No clear difference in IL-12 immunostaining was detected in epithelial cells (J) as compared to that of uninfected antrum mucosa (B). In contrast, a stronger immunostaining of p19 was observed in the foveolar epithelium and in immune cells including a lymph follicle (K). Ebi3 staining was only detected in the glandular neck zone of the antrum mucosa (L), similar to *H. pylori* negative mucosa (D). The corpus mucosa (M) contains increased immune cells (hematoxylin-eosin). No clear difference in IL-12 immunostaining was detected in epithelial cells (N) as compared to that of uninfected corpus mucosa (F). In contrast, a stronger immunostaining of p19 was observed in the foveolar epithelium, corpus glands with parietal cells, and immune cells (O). No Ebi3 staining was detected in the corpus mucosa (P). Scale bar=50µm.

**Figure 6:**
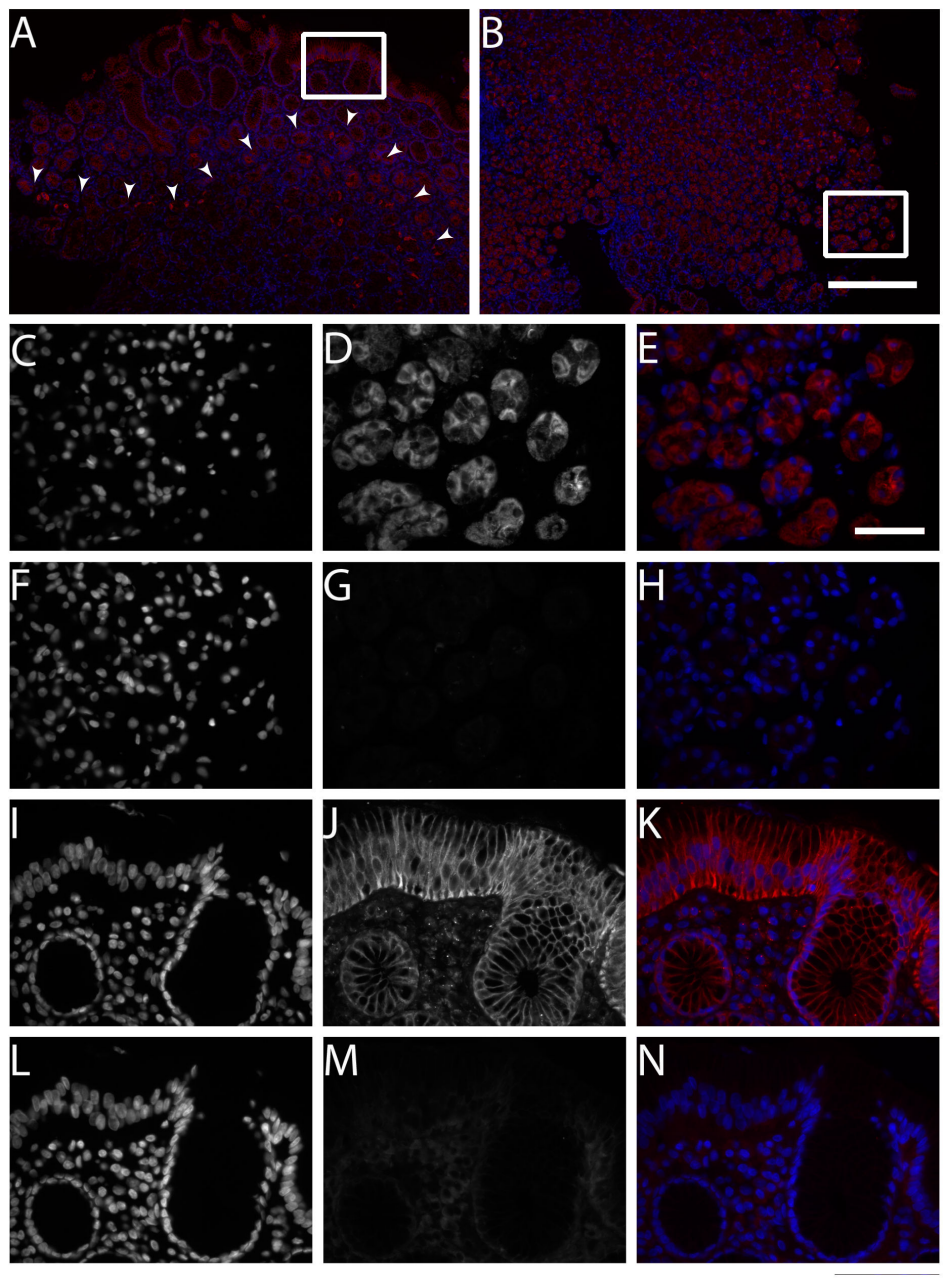
Immunofluorescence staining of p19 in human gastric mucosa. Immunofluorescence microscopic images depicting localization of p19 using a polyclonal anti-p19 antibody (red) in paraffin-embedded sections from antrum (A) and corpus (B) of *H. pylori* negative gastritis case. Both A and B are stitched overview images, a magnified region of interest (ROI) is indicated with a white square. Images (C-E) are the magnified ROI in A, whereas images (I-K) are the magnified ROI in B. Notably, gastric epithelial p19 expression showed two distinct staining patterns: membranous and cytoplasmic. The membranous was more clearly seen in the gastric superficial foveolar epithelium; the main site of host-bacteria interaction (J), whereas cytoplasmic pattern was mainly seen in the glandular compartment (D). As a function of fluorescence intensity, cells with stronger staining intensity were identified in the glandular neck zone of the antrum mucosa (A; arrowheads). Adjacent serial sections were used as negative controls to prove the specificity of the anti-p19 staining (F-H; L-N). Nuclei were stained with DAPI (blue). Scale bar (A-B)=250µm; (C-N)=50µm.

To examine protein expression of IL-12, p19, and Ebi3 in gastric biopsies of gastritis cases we used IHC (Figure 5). IL-12 was detected in gastric epithelial cells and immune cells in antrum and corpus mucosae of all specimens, irrespective of *H. pylori* status (Figure 5B, F, J, and N). Immunostaining of p19 was detected in both gastric epithelial cells and immune cells of all specimens. However, in the antrum, more staining intensity was observed in the superficial foveolar epithelium and in the glandular neck zone of the mucosa (Figure 5C and k), whereas in the corpus, both foveolar and glandular compartments were stained almost alike (Figure 5G and O). In contrast to IL-12 and Ebi3, increased immunostaining of p19 was observed in *H. pylori* gastritis (Figure 5K and O) compared to that of *H. pylori* negative mucosae of antrum and corpus (Figure 5C and G). In addition, higher amounts of p19*-*positive immune cells were detected in *H. pylori* gastritis. To better resolve protein localization of p19 in gastric mucosa we performed IF staining. Two distinct staining patterns could be identified: a membranous localization, which was more clearly seen in the superficial foveolar cells (Figure 6J), and a cytoplasmic localization, which was more obviously seen in the glands of the gastric corpus mucosa (Figure 6D). Interestingly, Ebi3 immunostaining was detected in the glandular neck part of the antrum (Figure 5D and L), but not in the corpus (Figure 5H and P). However, the staining pattern was almost the same between *H. pylori* positive (Figure 5L) and *H. pylori* negative gastritis cases (Figure 5D). 

## Discussion

In this study we report a differentially regulated epithelial-derived expression of *IL23A*, *EBI3*, and their receptors: *IL23R* and *IL6ST*, respectively with *H. pylori* infection. Since its discovery in 2000, p19 (encoded by *IL23A*) has long been known to be expressed by T cells, activated macrophages, and dendritic cells. It was also shown that the formation of biologically active IL-23 (p19/p40) heterodimer requires synthesis of both subunits (α and β chains) within the same cell [3]. The latter observation has been a typical propensity for the whole family of IL-12-related cytokines. Notably, epithelial cells are considered to be the initial site of contact between host and pathogens, and can play a pivotal role in innate mucosal response by expression of important inflammatory mediators (like IL-8, IL-6, and TNFα), or by presenting antigens to immune cells to promote adaptive immunity. Moreover, in gastric mucosa, epithelial cells outnumber the infiltrated immune cells, at least at early stages of infection, and so their contribution to express IL-12-related molecules should not be underestimated. Indeed, research to date has tended to focus on studying these molecules in immune cells rather than in epithelial cells, which are at the forefront of host’s response to infections. Therefore, we used AGS and MKN-28 cells in order to pinpoint the role of gastric epithelial cells alone in the expression of these molecules, based on the fact that these cell lines are of one cell type and devoid of immune cells. In Table 5 we provide a summary of our expression analysis versus a literature review of the currently known cell types to express these transcripts in human. In our study, the differentially regulated epithelial-derived expression of *IL23A* and *EBI3* with their receptors upon infection with different strains of *H. pylori* suggests a possible biological role for these molecules either alone or in combination with other molecules. In support of the latter hypothesis, it has been shown that an individual chain of IL-12 family could deliver a biological function like p28 (IL-30) [22], or a functional homodimer could be obtained by self-pairing like p40 (IL-12p80) [23], another added layer of complexity is the ability to bind other relatives of IL-6 superfamily of cytokines like p28 with cytokine like factor CLF [24]. A similar complexity has also been described for receptors of these chains making the exact biological function of these molecules enigmatic. Furthermore, the possibility of other combinations like (p19/Ebi3) and (p28/p40) cannot be excluded and can be physiologically relevant in favor of the previously described propensity of chain sharing. Interestingly, our analysis revealed a significant positive correlation between *IL23A* and *EBI3* after infection with P1 strain only (Figure 4B). It remains unclear whether this correlation implies an upregulated p19/Ebi3 heterodimer (IL-35) that might be elicited by certain virulence factor(s) exclusively expressed in P1 strain. In accordance with previous reports, our results showed a consistent induction of *IL8* by *H. pylori* infected gastric epithelial cells. Remarkably, both *IL8* and *IL23A* shared a pronounced and similar pattern of expression, in which the strongest alterations were seen with P1, whereas M2 infection or application of the filter cap after infection were associated with minimal alterations almost similar to that of controls (no *H. pylori*). This pattern was not evident for other regulated genes investigated in our study (*IL23R*, *EBI3*, and *IL6ST*). This further supports the importance of T4SS to mediate cellular alterations through translocation of CagA protein and other molecules into epithelial cells. It can be deduced that about half of the *IL23A* upregulation was attributed to CagA protein alone by comparing expression folds between wild-type (P1) and its CagA mutant (M1) (Table 2). However, small alterations in expression of both *IL8* and *IL23A* could still be seen with M2 infection or after application of the filter cap (in case of BCM-300 strain). From the latter observation, it can be inferred that T4SS and direct epithelial contact were not indispensable and secretory factors by *H. pylori*, independent of the secretion apparatus, might be the culprits for these small persistent alterations.

**Table 5:** Gene expression of IL-12-related transcripts in AGS and MKN-28 gastric epithelial cells versus the currently known cell types to express these transcripts in human from literature.

Transcript	AGS	MKN-28	Cell type^1^	Reference
***IL23A***	Yes	Yes	T, activated MΦ, DC, colon epithelium	[3,25,27]
***IL23R***	Yes	Yes	activated T, NK	[28,29]
***EBI3***	Yes	Yes	B, MΦ, trophoblast, colon epithelium	[30–32,25,33]
***IL6ST***	Yes	Yes	ubiquitous	[34,35]
***IL12A***	Yes	Yes	ubiquitous	[36–40]
***IL12RB2***	No	No	activated T, NK, B	[40–45]
***IL12B***	No	No	MΦ, DC, B, neutrophil	[36–40,46]
***IL12RB1***	No	No	T, NK, B	[41–43,47]
***IL27A***	No	No	MΦ, DC	[5]
***IL27RA***	Yes	Yes	T, NK, monocyte, mast cell	[48–50]

^1^ B; B lymphocyte, DC; dendritic cell, MΦ; macrophage, NK; natural killer cell, T; T lymphocyte

Another important observation was the detection of both *IL23A* and *IL12A* in gastric epithelial cells without the co-expression of *IL12B* (encodes p40), which is necessary to form functional IL-23 (p19/p40) and IL-12 (p35/p40), respectively. In a striking analogy to our findings, a study by Maaser et al. showed epithelial-derived expression and regulation of *IL23A*, *EBI3*, *IL12A*, but not *IL27A*, nor *IL12B* in human colon adenocarcinoma cell lines co-infected with *Salmonella* [25]. These analogous findings between gastric and colonic cell lines may denote a common, yet poorly understood innate epithelial defense mechanism against pathogens. In a different model, Schmidt et al. demonstrated an upregulation of *IL27A* in active Crohn’s disease (CD), but not in ulcerative colitis (UC). Likewise, upregulation of both *EBI3* and *IL27A* was only specific to CD. However, all transcripts of monomeric chains were detected in colonic mucosa except *IL12B* [26]. Although the latter two studies didn’t investigate transcript levels of receptors, we can generally hypothesize that IL-12-related transcripts may demonstrate a distinct expression profile that is specific to type of tissue and related pathologies.

As a proof-of-principle experiment, we performed IHC analysis for a subset of IL-12 family in gastric biopsies of gastritis cases with and without *H. pylori* infection. IL-12, p19, and Ebi3 were stained by IHC, whereas p19 was additionally stained by IF to study its localization. Nevertheless, investigating the remaining chains, cytokines, and corresponding chain receptors in context of *H. pylori* infection is strongly recommended for future research. As summarized in Table 6, immunostaining of the gastric mucosa confirmed our gene expression findings in gastric epithelial cell lines.

**Table 6:** Summary of the five IL-12 family chains and their expression in AGS, MKN-23 gastric epithelial cells and in human gastric mucosa as detected by RT-qPCR and immunohistochemistry, respectively.

**Transcript**	**RT-qPCR in AGS and MKN-28**	**Chain**	**Immunohistochemistry in gastric mucosa**	
	**Epithelial cells**		**Epithelial cells**	**Immune cells**
*IL23A*	Yes	p19	Yes	Yes
*EBI3*	Yes	Ebi3	Yes	No
*IL12A*	Yes	p35	Yes	Yes
*IL12B*	No	p40	?	?
*IL27A*	No	p28	-	-

IL-12 was found to be diffusely stained in gastric epithelial cells irrespective of *H. pylori* status (Figure 5B, F, J, and N). Since the used anti-IL-12 antibody gives signal by binding to the heterodimeric structure of IL-12 (IL-12p70) as well as to either of the monomeric chains (IL-12p35 or IL-12p40); therefore, the three interpretations of the result are possible. Nevertheless, immunostaining due to p35 chain alone is the mostly likely one, since its corresponding transcript, and not that of p40, was detected in our gene expression study. Immunostaining of p28 was discouraged as its transcript could not be detected by RT-qPCR. It is important to point out that the possibility of epithelial-derived expression of p40 is critical, and would imply the ability of epithelial cells to produce IL-12p70 and IL-23 cytokines upon pairing with p35 and p19, respectively. Consequently, these epithelial-derived cytokines could potentially drive T cell differentiation into Th1 or Th17 type of immune response. What opposes this possibility was the undetected gene expression of p40 transcript (*IL12B*) in gastric epithelial cell lines.

For Ebi3, which is a β chain known to be shared by two mainly anti-inflammatory cytokines: IL-27 (p28/Ebi3) and IL-35 (p35/Ebi3), it is interesting to see this molecule being only stained in the antrum; the primary habitat for *H. pylori* in the stomach. Moreover, the immunoreactivity of Ebi3 was clustered in the glandular neck zone of the antrum mucosa (Figure 5D and L); the same zone area where higher p19 immunoreactivity was detected (Figures 5C and 6A). Our gene expression analysis revealed induction of *EBI3* by *H. pylori* co-infected gastric cell lines, and correlation with *IL23A* expression after P1 strain infection (Figure 4B). Putting all findings together, one might speculate a functional interaction between these two α and β chains, at least in the antrum. The detected p35 immunoreactivity in the same area of the antrum would add another possibility of IL-35 (p35/Ebi3) release by the mucosa. Nevertheless, the function of Ebi3, whether in a monomeric or a heterodimeric structure, is an intriguing one and needs to be explored in further research.

Considering the effect of *H. pylori* infection on the immunoreactivity of IL-12 and Ebi3 in gastritis, our qualitative assessment of the selected cases revealed similar staining patterns regardless of *H. pylori* infection. However, to better examine this effect, more cases need to be evaluated and semi-quantitatively scored in order to reach a sound statistical power.

In support of our gene expression analysis, an increased immunoreactivity of p19 was observed in *H. pylori* gastritis (Figure 5K and O) compared to that of *H. pylori* negative gastritis (Figure 5C and G), suggesting upregulation of p19 by *H. pylori* in gastritis. It remains unclear, though, whether function of this chain differs with cell type, being an immune or an epithelial cell, and whether this function can be delivered by p19, as a monomeric, polymeric, or modified molecule. It has been estimated that more than 50% of all polypeptides are covalently modiﬁed by glycans; the most abundant and structurally diverse type of posttranslational modiﬁcation (PTM) occurring on proteins destined to be secreted or membrane-bound. A membranous localization of 19 was clearly demonstrated by our IF study, especially in the superficial foveolar epithelium (Figure 6J), which is the main site of host-bacterial interaction. Added to that, p19 protein is bioinformatically predicted to undergo mucin-type *O*-glycosylation, but not *N*-glycosylation. Therefore, it is tempting to speculate that modified p19 protein may contribute to the gastric epithelial glycocalyx. Glycan profiling of this molecule would possibly provide more in-depth knowledge about its function in context of *H. pylori* infection.

In a nutshell, the most obvious finding to emerge from this study is the contribution of gastric epithelial cells to molecules that belong to the IL-12 family of cytokines, which is against the dogma that these molecules are primarily produced by immune cells. Since epithelial cells are the predominant cell type in gastric mucosa, hence, these findings provide support for a conceptual premise that epithelial cells could have a major impact on the type of immune response at any given point via epithelial-derived immune mediators whose exact function remains elusive. Furthermore, our gene expression and IHC/IF studies were in agreement to identify the importance of p19 as the most relevant molecule in IL-12 family to *H. pylori* infection in terms of expression and localization. 

## References

[1] Vignali DAA, Kuchroo VK (2012) IL-12 family cytokines: immunological playmakers. Nat Immunol 13: 722–728. doi:10.1038/ni.2366. PubMed: 22814351. 22814351 10.1038/ni.2366 PMC4158817

[2] Korn T, Bettelli E, Oukka M, Kuchroo VK (2009) IL-17 and Th17 Cells. Annu Rev Immunol 27: 485–517. doi:10.1146/annurev.immunol.021908.132710. PubMed: 19132915. 19132915 10.1146/annurev.immunol.021908.132710

[3] Oppmann B, Lesley R, Blom B, Timans JC, Xu Y et al. (2000) Novel p19 protein engages IL-12p40 to form a cytokine, IL-23, with biological activities similar as well as distinct from IL-12. Immunity 13: 715–725. doi:10.1016/S1074-7613(00)00070-4. PubMed: 11114383. 11114383 10.1016/s1074-7613(00)00070-4

[4] Barnes MJ, Powrie F (2009) Regulatory T cells reinforce intestinal homeostasis. Immunity 31: 401–411. doi:10.1016/j.immuni.2009.08.011. PubMed: 19766083. 19766083 10.1016/j.immuni.2009.08.011

[5] Pflanz S, Timans JC, Cheung J, Rosales R, Kanzler H et al. (2002) IL-27, a heterodimeric cytokine composed of Ebi3 and p28 protein, induces proliferation of naive CD4+ T cells. Immunity 16: 779–790. doi:10.1016/S1074-7613(02)00324-2. PubMed: 12121660. 12121660 10.1016/s1074-7613(02)00324-2

[6] Wojno EDT, Hunter CA (2012) New directions in the basic and translational biology of interleukin-27. Trends Immunol 33: 91–97. doi:10.1016/j.it.2011.11.003. PubMed: 22177689. 22177689 10.1016/j.it.2011.11.003 PMC3273610

[7] Collison LW, Workman CJ, Kuo TT, Boyd K, Wang Y et al. (2007) The inhibitory cytokine IL-35 contributes to regulatory T-cell function. Nature 450: 566–569. doi:10.1038/nature06306. PubMed: 18033300. 18033300 10.1038/nature06306

[8] Collison LW, Vignali DAA (2008) Interleukin-35: odd one out or part of the family? Immunol Rev 226: 248–262. doi:10.1111/j.1600-065X.2008.00704.x. PubMed: 19161429. 19161429 10.1111/j.1600-065X.2008.00704.x PMC2631363

[9] Vignali DAA, Collison LW, Workman CJ (2008) How regulatory T cells work. Nat Rev Immunol 8: 523–532. doi:10.1038/nri2343. PubMed: 18566595. 18566595 10.1038/nri2343 PMC2665249

[10] Censini S, Lange C, Xiang Z, Crabtree JE, Ghiara P et al. (1996) *cag*, a pathogenicity island of *Helicobacter* *pylori*, encodes type I-specific and disease-associated virulence factors. Proc Natl Acad Sci U S A 93: 14648–14653. doi:10.1073/pnas.93.25.14648. PubMed: 8962108. 8962108 10.1073/pnas.93.25.14648 PMC26189

[11] Voth DE, Broederdorf LJ, Graham JG (2012) Bacterial type IV secretion systems: versatile virulence machines. Future Microbiol 7: 241–257. doi:10.2217/fmb.11.150. PubMed: 22324993. 22324993 10.2217/fmb.11.150 PMC3563059

[12] Covacci A, Rappuoli R (2000) Tyrosine-phosphorylated bacterial proteins. J Exp Med 191: 587–592. doi:10.1084/jem.191.4.587. PubMed: 10684850. 10684850 10.1084/jem.191.4.587 PMC2195833

[13] Stein M, Rappuoli R, Covacci A (2000) Tyrosine phosphorylation of the *Helicobacter* *pylori* CagA antigen after cag-driven host cell translocation. Proc Natl Acad Sci U S A 97: 1263–1268. doi:10.1073/pnas.97.3.1263. PubMed: 10655519. 10655519 10.1073/pnas.97.3.1263 PMC15590

[14] Backert S, Ziska E, Brinkmann V, Zimny-Arndt U, Fauconnier A et al. (2000) Translocation of the *Helicobacter* *pylori* CagA protein in gastric epithelial cells by a type IV secretion apparatus. Cell Microbiol 2: 155–164. doi:10.1046/j.1462-5822.2000.00043.x. PubMed: 11207572. 11207572 10.1046/j.1462-5822.2000.00043.x

[15] Malfertheiner P, Selgrad M, Wex T, Bornschein J, Emanuela P et al. (2012) Efficacy of an investigational recombinant antigen based vaccine against a CagA *H.* *pylori* infectious challenge in healthy volunteers (abstract). Gastroenterology 142 Suppl 1: S–184.

[16] Crabtree JE, Farmery SM, Lindley IJ, Figura N, Peichl P et al. (1994) CagA/cytotoxic strains of *Helicobacter* *pylori* and interleukin-8 in gastric epithelial cell lines. J Clin Pathol 47: 945–950. doi:10.1136/jcp.47.10.945. PubMed: 7962609. 7962609 10.1136/jcp.47.10.945 PMC502181

[17] Rho H-W, Lee B-C, Choi E-S, Choi I-J, Lee Y-S et al. (2010) Identification of valid reference genes for gene expression studies of human stomach cancer by reverse transcription-qPCR. BMC Cancer 10: 240. doi:10.1186/1471-2407-10-240. PubMed: 20507635. 20507635 10.1186/1471-2407-10-240 PMC2887403

[18] Vandesompele J, De Preter K, Pattyn F, Poppe B, Van Roy N et al. (2002) Accurate normalization of real-time quantitative RT-PCR data by geometric averaging of multiple internal control genes. Genome Biol 3: 1–12. PubMed: 12184808. 10.1186/gb-2002-3-7-research0034 PMC126239 12184808

[19] Kohl Matthias (2007) SLqPCR: Functions for analysis of real-time quantitative PCR data at SIRS-Lab GmbH. R package, SIRS-Lab GmbH. Jena

[20] Core R Team (2012) R: A Language and Environment for Statistical Computing. Vienna, Austria: R Foundation for Statistical Computing.

[21] Wickham H (2009) ggplot2: Elegant Graphics for Data. Analysis. 2 ^nd^ Printing. Springer Verlag . 221 p

[22] Stumhofer JS, Tait ED iii; Quinn WJ, Hosken N, Spudy B et al. (2010) A role for IL-27p28 as an antagonist of gp130-mediated signaling. Nat Immunol 11: 1119–1126. doi:10.1038/ni.1957. PubMed: 21057510. doi:10.1038/ni.1957 PubMed: 21057510 21057510 10.1038/ni.1957 PMC3059498

[23] Mattner F, Fischer S, Guckes S, Jin S, Kaulen H et al. (1993) The interleukin-12 subunit p40 specifically inhibits effects of the interleukin-12 heterodimer. Eur J Immunol 23: 2202–2208. doi:10.1002/eji.1830230923. PubMed: 8103745. 8103745 10.1002/eji.1830230923

[24] Crabé S, Guay-Giroux A, Tormo AJ, Duluc D, Lissilaa R et al. (2009) The IL-27 p28 subunit binds cytokine-like factor 1 to form a cytokine regulating NK and T Cell activities requiring IL-6R for signaling. J Immunol 183: 7692–7702. doi:10.4049/jimmunol.0901464. PubMed: 19933857. 19933857 10.4049/jimmunol.0901464

[25] Maaser C, Egan LJ, Birkenbach MP, Eckmann L, Kagnoff MF (2004) Expression of Epstein–Barr virus-induced gene 3 and other interleukin-12-related molecules by human intestinal epithelium. Immunology 112: 437–445. doi:10.1111/j.1365-2567.2004.01895.x. PubMed: 15196212. 15196212 10.1111/j.1365-2567.2004.01895.x PMC1782502

[26] Schmidt C, Giese T, Ludwig B, Mueller-Molaian I, Marth T et al. (2005) Expression of interleukin-12-related cytokine transcripts in inflammatory bowel disease: elevated interleukin-23p19 and interleukin-27p28 in Crohn’s disease but not in ulcerative colitis. Inflamm Bowel Dis 11: 16–23. doi:10.1097/00054725-200501000-00003. PubMed: 15674109. 15674109 10.1097/00054725-200501000-00003

[27] McKenzie BS, Kastelein RA, Cua DJ (2006) Understanding the IL-23-IL-17 immune pathway. Trends Immunol 27: 17–23. doi:10.1016/j.it.2005.10.003. PubMed: 16290228. 16290228 10.1016/j.it.2005.10.003

[28] Parham C, Chirica M, Timans J, Vaisberg E, Travis M et al. (2002) A Receptor for the heterodimeric cytokine IL-23 is composed of IL-12Rβ1 and a novel cytokine receptor subunit, IL-23R. J Immunol 168: 5699–5708. PubMed: 12023369. 12023369 10.4049/jimmunol.168.11.5699

[29] Maul J, Zeitz M (2012) Ulcerative colitis: immune function, tissue fibrosis and current therapeutic considerations. Langenbecks Arch Surg 397: 1–10. doi:10.1007/s00423-011-0789-4. PubMed: 21479621. 21479621 10.1007/s00423-011-0789-4

[30] Devergne O, Hummel M, Koeppen H, Le Beau, Nathanson EC et al. (1996) A novel interleukin-12 p40-related protein induced by latent Epstein-Barr virus infection in B lymphocytes. J Virol 70: 1143–1153. PubMed: 8551575. 8551575 10.1128/jvi.70.2.1143-1153.1996 PMC189923

[31] Eckmann L, Smith JR, Housley MP, Dwinell MB, Kagnoff MF (2000) Analysis by high density cDNA arrays of altered gene expression in human intestinal epithelial cells in response to infection with the invasive enteric bacteria *Salmonella* . J Biol Chem 275: 14084–14094. doi:10.1074/jbc.275.19.14084. PubMed: 10799483. 10799483 10.1074/jbc.275.19.14084

[32] Devergne O, Coulomb-L’Herminé A, Capel F, Moussa M, Capron F (2001) Expression of Epstein-Barr virus-induced gene 3, an interleukin-12 p40-related molecule, throughout human pregnancy: involvement of syncytiotrophoblasts and extravillous trophoblasts. Am J Pathol 159: 1763–1776. doi:10.1016/S0002-9440(10)63023-4. PubMed: 11696437. 11696437 10.1016/S0002-9440(10)63023-4 PMC1867066

[33] Li X, Mai J, Virtue A, Yin Y, Gong R et al. (2012) IL-35 is a novel responsive anti-inflammatory cytokine — a new system of categorizing anti-inflammatory cytokines. PLOS ONE 7: 1–13. 10.1371/journal.pone.0033628 PMC3306427 22438968

[34] Taga T, Hibi M, Hirata Y, Yamasaki K, Yasukawa K et al. (1989) Interleukin-6 triggers the association of its receptor with a possible signal transducer, gp130. Cell 58: 573–581. doi:10.1016/0092-8674(89)90438-8. PubMed: 2788034. 2788034 10.1016/0092-8674(89)90438-8

[35] Müller-Newen G (2003) The cytokine receptor gp130: faithfully promiscuous. Sci Signal, 2003: 2003: 1–3 PubMed: 14506288. 10.1126/stke.2003.201.pe40 14506288

[36] Stern AS, Podlaski FJ, Hulmes JD, Pan YC, Quinn PM et al. (1990) Purification to homogeneity and partial characterization of cytotoxic lymphocyte maturation factor from human B-lymphoblastoid cells. Proc Natl Acad Sci U S A 87: 6808–6812. doi:10.1073/pnas.87.17.6808. PubMed: 2204066. 2204066 10.1073/pnas.87.17.6808 PMC54627

[37] Wolf SF, Temple PA, Kobayashi M, Young D, Dicig M et al. (1991) Cloning of cDNA for natural killer cell stimulatory factor, a heterodimeric cytokine with multiple biologic effects on T and natural killer cells. J Immunol 146: 3074–3081. PubMed: 1673147. 1673147

[38] Bliss SK, Butcher BA, Denkers EY (2000) Rapid recruitment of neutrophils containing prestored IL-12 during microbial infection. J Immunol 165: 4515–4521. PubMed: 11035091. 11035091 10.4049/jimmunol.165.8.4515

[39] Liu J, Cao S, Kim S, Chung EY, Homma Y et al. (2005) Interleukin-12: an update on its immunological activities, signaling and regulation of gene expression. Curr. Immunol Rev 1: 119–137. doi:10.2174/1573395054065115. 10.2174/1573395054065115 PMC2965603 21037949

[40] Airoldi I, Guglielmino R, Carra G, Corcione A, Gerosa F et al. (2002) The interleukin-12 and interleukin-12 receptor system in normal and transformed human B lymphocytes. Haematologica 87: 434–442. PubMed: 11940489. 11940489

[41] Chizzonite R, Truitt T, Desai BB, Nunes P, Podlaski FJ et al. (1992) IL-12 receptor. I. Characterization of the receptor on phytohemagglutinin-activated human lymphoblasts. J Immunol 148: 3117–3124.. 1578138

[42] Desai BB, Quinn PM, Wolitzky AG, Mongini PK, Chizzonite R et al. (1992) IL-12 receptor. II. Distribution and regulation of receptor expression. J Immunol 148: 3125–3132.. 1578139

[43] Presky DH, Yang H, Minetti LJ, Chua AO, Nabavi N et al. (1996) A functional interleukin 12 receptor complex is composed of two β-type cytokine receptor subunits. Proc Natl Acad Sci U S A 93: 14002–14007. doi:10.1073/pnas.93.24.14002. PubMed: 8943050. 8943050 10.1073/pnas.93.24.14002 PMC19484

[44] Rogge L, Barberis-Maino L, Biffi M, Passini N, Presky DH et al. (1997) Selective expression of an interleukin-12 receptor component by human T helper 1 cells. J Exp Med 185: 825–832. doi:10.1084/jem.185.5.825. PubMed: 9120388. 9120388 10.1084/jem.185.5.825 PMC2196163

[45] Szabo SJ, Dighe AS, Gubler U, Murphy KM (1997) Regulation of the interleukin (IL)-12R β2 subunit expression in developing T helper 1 (Th1) and Th2 cells. J Exp Med 185: 817–824. doi:10.1084/jem.185.5.817. PubMed: 9120387. 9120387 10.1084/jem.185.5.817 PMC2196166

[46] Ma X, Chow JM, Gri G, Carra G, Gerosa F et al. (1996) The interleukin 12 p40 gene promoter is primed by interferon gamma in monocytic cells. J Exp Med 183: 147–157. doi:10.1084/jem.183.1.147. PubMed: 8551218. 8551218 10.1084/jem.183.1.147 PMC2192398

[47] Wu C, Warrier RR, Wang X, Presky DH, Gately MK (1997) Regulation of interleukin-12 receptor beta1 chain expression and interleukin-12 binding by human peripheral blood mononuclear cells. Eur J Immunol 27: 147–154. doi:10.1002/eji.1830270122. PubMed: 9022011. 9022011 10.1002/eji.1830270122

[48] Sprecher CA, Grant FJ, Baumgartner JW, Presnell SR, Schrader SK et al. (1998) Cloning and characterization of a novel class I cytokine receptor. Biochem Biophys Res Commun 246: 82–90. doi:10.1006/bbrc.1998.8576. PubMed: 9600072. 9600072 10.1006/bbrc.1998.8576

[49] Chen Q, Ghilardi N, Wang H, Baker T, Xie M-H et al. (2000) Development of Th1-type immune responses requires the type I cytokine receptor TCCR. Nature 407: 916–920. doi:10.1038/35038103. PubMed: 11057672. 11057672 10.1038/35038103

[50] Pflanz S, Hibbert L, Mattson J, Rosales R, Vaisberg E et al. (2004) WSX-1 and glycoprotein 130 constitute a signal-transducing receptor for IL-27. J Immunol 172: 2225–2231. PubMed: 14764690. 14764690 10.4049/jimmunol.172.4.2225

